# ^18^F-Florzolotau PET imaging captures the distribution patterns and regional vulnerability of tau pathology in progressive supranuclear palsy

**DOI:** 10.1007/s00259-022-06104-0

**Published:** 2023-01-11

**Authors:** Feng-Tao Liu, Jia-Ying Lu, Xin-Yi Li, Xiao-Niu Liang, Fang-Yang Jiao, Jing-Jie Ge, Ping Wu, Gen Li, Bo Shen, Bin Wu, Yi-Min Sun, Yu-Hua Zhu, Jian-Feng Luo, Tzu-Chen Yen, Jian-Jun Wu, Chuan-Tao Zuo, Jian Wang, for the Progressive Supranuclear Palsy Neuroimage Initiative (PSPNI)

**Affiliations:** 1 Department of Neurology, National Clinical Research Center for Aging and Medicine & National Center for Neurological Disorders, Huashan Hospital, Fudan University, 12 Middle Wulumuqi Road, Shanghai, 200040 China; 2 Department of Nuclear Medicine & PET Center, National Clinical Research Center for Aging and Medicine, & National Center for Neurological Disorders, Huashan Hospital, Fudan University, 518 East Wuzhong Road, Shanghai, 200235 China; 3 Department of Biostatistics, School of Public Health, Fudan University, Shanghai, China; 4 APRINOIA Therapeutics Co., Ltd, Suzhou, China; 5 Human Phenome Institute, Fudan University, Shanghai, China

**Keywords:** ^18^F-Florzolotau, Tau, Positron emission tomography, Progressive supranuclear palsy

## Abstract

**Purpose:**

Human post mortem studies have described the topographical patterns of tau pathology in progressive supranuclear palsy (PSP). Recent advances in tau PET tracers are expected to herald the next era of PSP investigation for early detection of tau pathology in living brains. This study aimed to investigate whether ^18^F-Florzolotau PET imaging may capture the distribution patterns and regional vulnerability of tau pathology in PSP, and to devise a novel image-based staging system.

**Methods:**

The study cohort consisted of 148 consecutive patients with PSP who had undergone ^18^F-Florzolotau PET imaging. The PSP rating scale (PSPrs) was used to measure disease severity. Similarities and differences of tau deposition among different clinical phenotypes were examined at the regional and voxel levels. An ^18^F-Florzolotau pathological staging system was devised according to the scheme originally developed for post mortem data. In light of conditional probabilities for the sequence of events, an ^18^F-Florzolotau modified staging system by integrating clusters at the regional level was further developed. The ability of ^18^F-Florzolotau staging systems to reflect disease severity in terms of PSPrs score was assessed by analysis of variance.

**Results:**

The distribution patterns of ^18^F-Florzolotau accumulation in living brains of PSP showed a remarkable similarity to those reported in post mortem studies, with the binding intensity being markedly higher in Richardson’s syndrome. Moreover, ^18^F-Florzolotau PET imaging allowed detecting regional vulnerability and tracking tau accumulation in an earlier fashion compared with post mortem immunostaining. The ^18^F-Florzolotau staging systems were positively correlated with clinical severity as reflected by PSPrs scores.

**Conclusions:**

^18^F-Florzolotau PET imaging can effectively capture the distribution patterns and regional vulnerability of tau pathology in PSP. The ^18^F-Florzolotau modified staging system holds promise for early tracking of tau deposition in living brains.

**Supplementary Information:**

The online version contains supplementary material available at 10.1007/s00259-022-06104-0.

## Introduction

Progressive supranuclear palsy (PSP) is a rare tauopathy in which the 4-repeat (4R) form of tau predominates [1]. Human post mortem studies have recently described the topographical patterns of tau pathology in PSP, from which a model of regional vulnerability can be developed [2]. Based on these results, a pathological staging scheme defining a temporal-spatial progression of PSP has been proposed [2] and independently validated in vivo [3]. Previous studies have shown that a straightforward correlation exists between the clinical severity of PSP and the underlying post mortem tau pathology [4, 5]. In this scenario, useful clinical information — including disease phenotyping and disease severity — can be gleaned from in vivo imaging biomarkers of tau.

Previous positron emission tomography (PET) studies focusing on the first-generation tau tracer ^18^F-Flortaucipir failed to capture pathological changes associated with PSP [6]. In addition, PET-derived indices were not clinically useful for predicting the disease course at the individual level [6]. These results can be attributed, at least in part, to off-target tracer binding and/or the inability of ^18^F-Flortaucipir to recognize 4R tau deposits [7]. However, recent advances in the field of PET tau tracers are expected to herald the next era of PSP investigation for early detection of 4R tau pathology in living brains. We previously reported the pilot observation of the pallido-nigro-luysian axis-dominant ^18^F-Florzolotau (a second-generation tau tracer also termed ^18^F-APN-1607 or ^18^F-PM-PBB3) uptake in a small cohort of clinically diagnosed patients with PSP [8], indicating the promising clinical utility of this imaging modality. This study was therefore undertaken to further explore the potential value of PET imaging with ^18^F-Florzolotau on a large cohort of patients with PSP across different phenotypes, and to investigate whether this imaging biomarker may capture the distribution patterns and regional vulnerability of tau pathology in PSP.

## Methods

### Participants

**Fig. 1:**
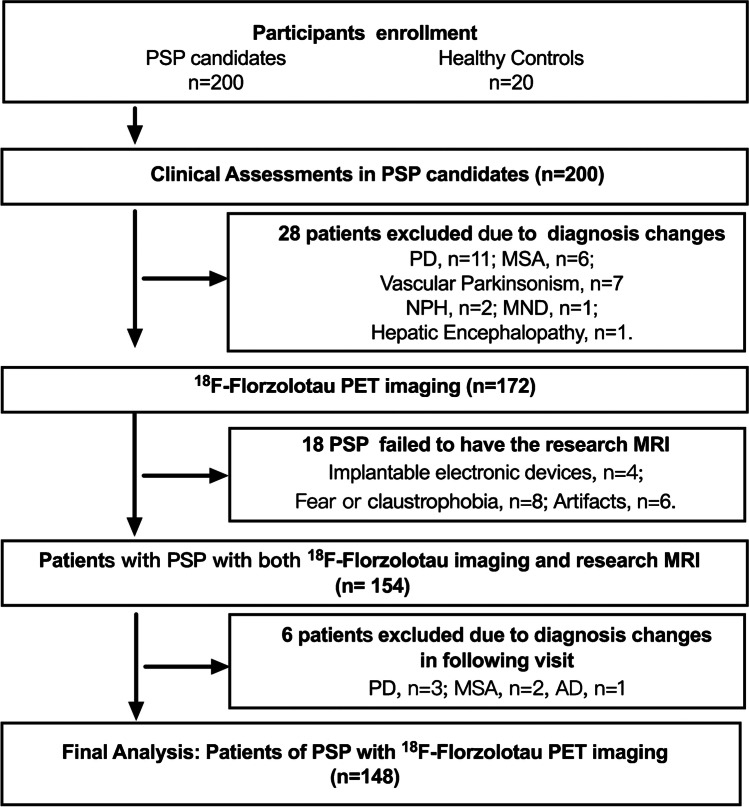
Flow of patients through the study. Abbreviations: PSP, progressive supranuclear palsy; PD, Parkinson’s disease; MSA, multiple system atrophy; NPH, normal pressure hydrocephalus; MND, motor neuron disease. Changes made to clinical diagnoses were implemented by an expert panel after reaching a consensus

All visits and procedures occurred at the Movement Disorders Clinic, Department of Neurology, Huashan Hospital, Fudan University (Shanghai, China) between May 2019 and January 2022. A total of 200 consecutive patients who met the Movement Disorder Society diagnostic criteria for PSP [9] were included. Patients with evidence of severe leukoencephalopathy or brain structural abnormalities on MRI were excluded. After formal recruitment, all participants underwent PET imaging. The study flow chart (Fig. 1) shows details about inclusion and exclusion of cases. Twenty subjects with a negative history for neurological or psychiatric disorders were included in the healthy control (HC) group. All participants provided written informed consent in accordance with the Helsinki declaration, and approval was granted by the Institutional Review Board of the Huashan Hospital (identifiers: KY2019-284, KY2019-433, and KY2020-1160).

### Clinical assessment

Patients with PSP were assessed after at least 12 h from the last dose of any antiparkinsonian medication (if used). Disease severity was characterized with the PSP rating scale (PSPrs). The Mini-Mental State Examination (MMSE) was used to test cognitive functioning. The L-dopa equivalent daily dose (LEDD) was calculated for each patient.

### Imaging acquisition

All eligible participants underwent ^18^F-Florzolotau tau PET imaging and T1-weighted MR imaging within 2 weeks from the baseline visit. The tosylate precursor of the tracer was obtained from APRINOIA Therapeutics (Suzhou, China). Because of its light sensitivity, both manufacturing and injection of ^18^F-Florzolotau were carried out under a green light-emitting diode (510 nm) [10]. ^18^F-Florzolotau tau PET imaging was acquired using a 20-min protocol (90 − 110 min) on a Biograph mCT Flow PET/CT system (Siemens Healthcare GmbH, Erlangen, Germany) and reconstructed using a 3D-ordered subset expectation maximization algorithm. T1-weighted MR imaging was acquired using a 3.0-T horizontal magnet (Discovery MR750; GE Medical Systems, Milwaukee, WI, USA). The details for imaging acquisition could be found in the previous publication [11].

### Image processing

Individual ^18^F-Florzolotau PET scans were resampled in the common space of the corresponding T1-weighted images and subsequently linearly and non-linearly registered to the Montreal Neurological Institute (MNI) brain atlas based on the transformation of individual T1-weighted images to the MNI space. Spatially normalized PET images were smoothed using a Gaussian kernel (full-width at half-maximum: 6 mm). The cerebellar grey matter was chosen as reference for intensity normalization [11]. All image processing was computed using Statistical Parametric Mapping (version 12) implemented in MATLAB 9.5 (MathWorks, Natick, MA, USA).

### Regions of interest and criteria for assessing tau deposition

The regions of interest (ROIs) for calculation of standardized uptake value ratios (SUVRs) were identified using the MNI PD25 template [12], the automated anatomical atlas three (AAL3) template [13], the merged AAL-VOI template in the PMOD software (http://www.pmod.com) as well as the templated generated from the Wake Forest University PickAtlas [14]. The following 18 subcortical ROIs were selected: bilateral regions of putamen, globus pallidus, thalamus, subthalamic nucleus, red nucleus, substantia nigra, raphe nuclei, locus coeruleus, dentate nucleus, and cerebellar white matter. We also examined a set of eight cortical ROIs (bilateral frontal, parietal, temporal, and occipital lobes). Mean SUVRs extracted from bilateral ROIs were used for subsequent analyses, with left and right SUVRs analyzed separately in the assignment of disease stages. Regional SUVR Z scores were calculated with the following formula: [(individual SUVR — mean SUVR in HCs)/standard deviation of the SUVR in HCs]. As for regional positivity, although a Z score ≥ 2 was generally recommended as a binary positivity criterion in previous publications in this field [10, 14], to detect early tau accumulation, we exploratively modified and extended the classification as follows:  < 1, negative (−);  ≥ 1 and  < 1.5, mildly positive (+);  ≥ 1.5 and  < 2, moderately positive (+ +); and  ≥ 2, strongly positive (+ + +).

### Conditional probability analysis

According to a recently described methodology [2], we applied conditional probability analysis to determine the patterns of tau accumulation in different anatomical regions. We initially assigned a score of 1 for strongly positive regions (SUVR Z score  ≥ 2) and a score of 0 for negative, mildly positive, or moderately positive regions (SUVR Z score  < 2) according to the commonly used dichotomization [11, 15]. The null hypothesis was that the probability of region A being positive (score = 1) while region B being negative (score = 0) and the region A being negative (score = 0) and region B being positive (score = 1) would be equally likely; thus, the A and B regions were considered to be simultaneously affected. This approach allowed assigning a conditional probability that one region would be affected by tau accumulation before another one. The McNemar’s test was applied to measure the strength of the evidence against the null hypothesis. The conditional probability was calculated using the crosstab function as previously described in SPSS for Windows (version 22.0; IBM, Armonk, NY, USA) [2]. If the conditional probability for one region was  ≥ 0.20 and the *p* value was < 0.01, the region was considered as most likely affected before the other, as previously reported [2]. The procedures used for calculation are described in the Supplementary Methods.

### ^18^F-Florzolotau PET staging systems

An ^18^F-Florzolotau pathological staging system was devised according to the scheme proposed in a recent post mortem study [2]. This was based on the analysis of a predefined set of ROIs in which the presence of tau pathology, as reflected by Z scores, was denoted as “ + ” (with “ + ”, “ +  + ”, and “ +  +  + ” reflecting mild, moderate, and strong involvement, respectively) and the absence by “ − ”. According to the ^18^F-Florzolotau pathological staging system, stage I − II was defined as evidence of ^18^F-Florzolotau binding in the globus pallidus, subthalamic nucleus, and putamen; stage III − IV as ^18^F-Florzolotau accumulation in the frontal lobe, dentate nucleus, and cerebral white matter; and stage V − VI as ^18^F-Florzolotau binding in the occipital lobe. In light of conditional probabilities for the sequence of events, and taking into account the evidence from the post mortem study [2], an ^18^F-Florzolotau modified staging system through the integration of clusters at the regional level was devised, focusing more on early tau accumulation.

### Statistics

All continuous variables were assessed for normality using the Kolmogorov–Smirnov test. Data were expressed as means ± standard deviations or medians and interquartile ranges for normally distributed and skewed variables, respectively. Differences were compared using Student’s *t*-tests and Mann–Whitney *U*, as appropriate. For comparisons of more than two groups, Gaussian variables were analyzed with one-way analysis of variance (ANOVA), whereas the Kruskal–Wallis test was used when data deviated from a normal distribution. Adjustment for multiple testing was performed using the conservative Bonferroni’s correction. Proportions were compared between groups using chi-square tests. A generalized linear model (GLM) adjusted for age, sex, and disease duration (if applicable) was used to estimate the intergroup differences in semiquantitative PET parameters of different target regions, with the Benjamini–Hochberg procedure applied to correct for multiple comparisons. ROI- and voxel-level statistical analyses were used to compare different PSP phenotypes on ^18^F-Florzolotau retention. A GLM was employed in ROI-level analysis. For voxel-level analysis, ^18^F-Florzolotau SUVR images were compared between groups after adjustment for age, sex, and disease duration (if applicable) using the two-sample *t*-test model provided by Statistical Parametric Mapping (version 12). Results were considered significant at a voxel-wise *p* < 0.001 (uncorrected) and a cluster-wise *p* < 0.05 (false discovery rate correction for multiple comparisons). One-way ANOVA was used for comparisons of disease severity between groups followed by Bonferroni’s post hoc tests. Differences concerning PSPrs scores across various disease stages were tested using one-way ANOVA. Statistical analyses were performed using SPSS for Windows (version 22.0; IBM, Armonk, NY, USA), unless otherwise indicated. All hypothesis testing was two-tailed, with statistical significance defined as a *p* value < 0.05, unless otherwise indicated.

## Results

### Patient characteristics

Among the 148 patients with PSP included in the study, 56.8% (*n* = 84) had probable PSP-Richardson’s syndrome (RS) and the remainder (*n* = 64, 43.2%) other PSP phenotypes (collectively termed PSP-non-RS). The general characteristics of patients with PSP are presented in Table 1 and Supplementary Table 1. Patients with PSP-RS differed from the remainder in having more severe disease (PSPrs scores, *p* < 0.01) and cognitive impairment (MMSE scores, *p* < 0.01). Data concerning HCs are summarized in Table 1.

**Table 1:** General characteristics of the study participants

	Entire cohort of patients with PSP	PSP-RS	PSP-non-RS	Healthy controls	p*	p^#^
Number of subjects	148	84	64	20	−	−
Sex (men/women)	83/65	43/41	40/24	6/14	0.03^a^	0.17^a^
Age (years)	65.7 ± 6.8	66.3 ± 5.8	65.0 ± 7.8	56.7 ± 7.3	< 0.01^b^	0.24^b^
Age at onset (years)	62.0 ± 7.1	63.0 ± 6.2	60.8 ± 8.0	N.A	−	0.07^b^
Disease duration (months)	38.0 (22.0,60.0)	38.0 (24.0, 52.0)	42.0 (18.0, 72.0)	N.A	−	0.46^c^
PSPrs score	28.0 (20.0, 37.5)	32.0 (22.0, 43.0)	23.5 (17.0, 31.0)	N.A	−	< 0.01^c^
MMSE score	25 (21, 27)	24 (19, 27)	26 (24, 28)	28 (27, 29)	< 0.01^c^	< 0.01^c^
Education (years)	9.0 (8.0, 12.0)	9.0 (6.0, 12.0)	11.0 (8.0, 12.3)	12.0 (9.0,15.8)	0.01^c^	0.12^c^
LEDD (mg)	400.0 (150.0, 600.0)	400.0 (150.0, 600.0)	400.0 (225.0, 650.0)	N.A	−	0.65^c^

Data are expressed as means ± standard deviations or medians (interquartile ranges), unless otherwise state. Abbreviations: *PSP*, progressive supranuclear palsy; *PSP-RS*, progressive supranuclear palsy-Richardson’s syndrome; *PSP-non-RS*, other PSP subtypes with the exception of progressive supranuclear palsy-Richardson’s syndrome; *PSPrs*, progressive supranuclear palsy rating scale; *MMSE*, Mini-Mental State Examination; *LEDD*, L-dopa equivalent daily dose; *N.A.*, not applicable

^*^ Patients with PSP *versus* healthy controls; ^#^ Patients with PSP-RS *versus* patients with PSP-non-RS

^a^ Chi-square test; ^b^ Student’s *t*-test; ^c^ Mann–Whitney *U* test

### Semi-quantitative analysis of ^18^F-Florzolotau binding

**Fig. 2:**
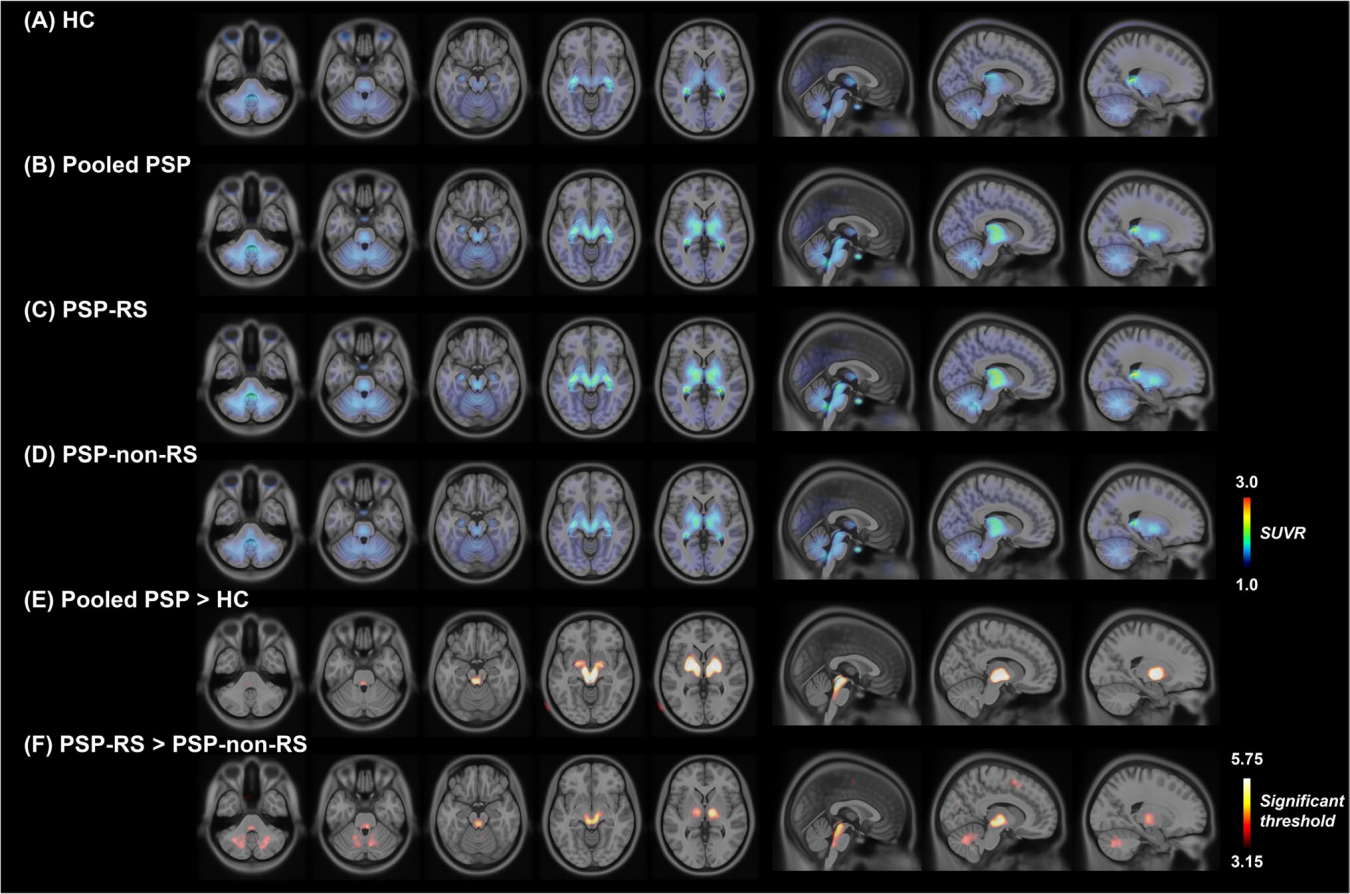
^18^F-Florzolotau PET images of patients with progressive supranuclear palsy. The images illustrate ^18^F-Florzolotau uptake in the HC group (panel A), entire patient cohort (panel B), as well as in the PSP-RS (panel C) and PSP-non-RS (panel D) groups. Compared with the HC group, patients with PSP showed significantly increased binding mainly in the pallido-nigro-luysian axis (panel E). While the patterns of tracer accumulation were similar in the two patient groups, the binding intensity was markedly lower in patients with PSP-non-RS (panel F). The color bars in panels A − D indicate SUVR values, whereas the color bar in panels E–F denote threshold values from the voxel-wise comparisons. Abbreviations: HC, healthy control; PSP, progressive supranuclear palsy; PSP-RS, progressive supranuclear palsy-Richardson’s syndrome; PSP-non-RS, other PSP subtypes expect progressive supranuclear palsy-Richardson’s syndrome; SUVR, standardized uptake value ratio

Figure 2 shows the mean summed ^18^F-Florzolotau PET images obtained from patients with PSP-RS, PSP-non-RS and HCs. The patterns of ^18^F-Florzolotau retention were similar in these two patient groups. However, voxel- (Fig. 2F, Supplementary Table 2) and regional-level (Table 2) analyses consistently found that the binding intensity was markedly lower in the PSP-non-RS group. Detailly, patients with PSP-RS had higher mean ^18^F-Florzolotau SUVR in the globus pallidus, subthalamic nucleus, red nucleus, substantia nigra, raphe nuclei, locus coeruleus, dentate nucleus, and cerebral white matter than patients with PSP-non-RS.

**Table 2:** Standardized uptake value ratio values in healthy controls, patients with progressive supranuclear palsy-Richardson’s syndrome, and other disease phenotypes

	PSP-RS (*n* = 84)	PSP-non-RS (*n* = 64)	HCs (*n* = 20)	PSP-RS *versus* PSP-non-RS, p^a^	PSP-RS *versus* HCs, p^b^	PSP-non-RS *versus* HCs p^b^
Frontal cortex	0.91 ± 0.10	0.89 ± 0.08	0.89 ± 0.07	0.40	0.90	0.78
Parietal cortex	0.97 ± 0.10	0.94 ± 0.09	0.93 ± 0.05	0.16	0.77	0.67
Temporal cortex	1.03 ± 0.12	1.00 ± 0.09	1.00 ± 0.06	0.35	0.65	0.90
Occipital cortex	1.10 ± 0.11	1.08 ± 0.09	1.08 ± 0.06	0.33	0.90	0.77
Putamen	1.29 ± 0.17	1.25 ± 0.14	1.14 ± 0.12	0.08	< 0.01	< 0.01
Globus pallidus	1.62 ± 0.21	1.54 ± 0.22	1.26 ± 0.12	0.01	< 0.01*	< 0.01*
Thalamus	1.66 ± 0.28	1.53 ± 0.25	1.41 ± 0.16	0.01	0.03	0.15
Subthalamic nucleus	1.86 ± 0.29	1.69 ± 0.26	1.34 ± 0.13	< 0.01*	< 0.01*	< 0.01*
Red nucleus	1.77 ± 0.30	1.62 ± 0.25	1.34 ± 0.09	< 0.01	< 0.01*	< 0.01*
Substantia nigra	1.54 ± 0.20	1.46 ± 0.21	1.29 ± 0.14	< 0.01	< 0.01*	< 0.01
Raphe nuclei	1.74 ± 0.28	1.57 ± 0.27	1.31 ± 0.11	< 0.01*	< 0.01*	< 0.01*
Locus coeruleus	1.47 ± 0.19	1.36 ± 0.17	1.26 ± 0.14	< 0.01*	< 0.01*	< 0.01
Dentate nucleus	1.47 ± 0.20	1.36 ± 0.15	1.32 ± 0.12	< 0.01	0.13	0.35
Cerebellar white matter	1.35 ± 0.13	1.28 ± 0.11	1.26 ± 0.09	< 0.01	0.20	0.69

^a^ A generalized linear model adjusted for age, sex, and disease duration was used to estimate intergroup differences related to semiquantitative PET parameters in different target regions; in all analyses, the Benjamini–Hochberg procedure was applied to correct for multiple comparisons

^b^ A generalized linear model adjusted for age and sex was used to estimate intergroup differences related to semiquantitative PET parameters in different target regions; in all analyses, the Benjamini–Hochberg procedure was applied to correct for multiple comparisons

Abbreviations: *PSP-RS*, progressive supranuclear palsy-Richardson’s syndrome; *PSP-non-RS*, other PSP subtypes with the exception of progressive supranuclear palsy-Richardson’s syndrome; *HCs*, healthy controls. * *p* < 0.0001

Consistent results were found when dividing the PSP-non-RS group into subgroups according to disease phenotypes [PSP with predominant gait freezing (PSP-PGF), PSP with predominant parkinsonism (PSP-P)] and comparing each phenotype individually with PSP-RS. No significant differences were seen between patients with PSP-PGF and PSP-P. The details are shown in Supplementary Table 2–3, and Supplementary Fig. 1.

**Fig. 3:**
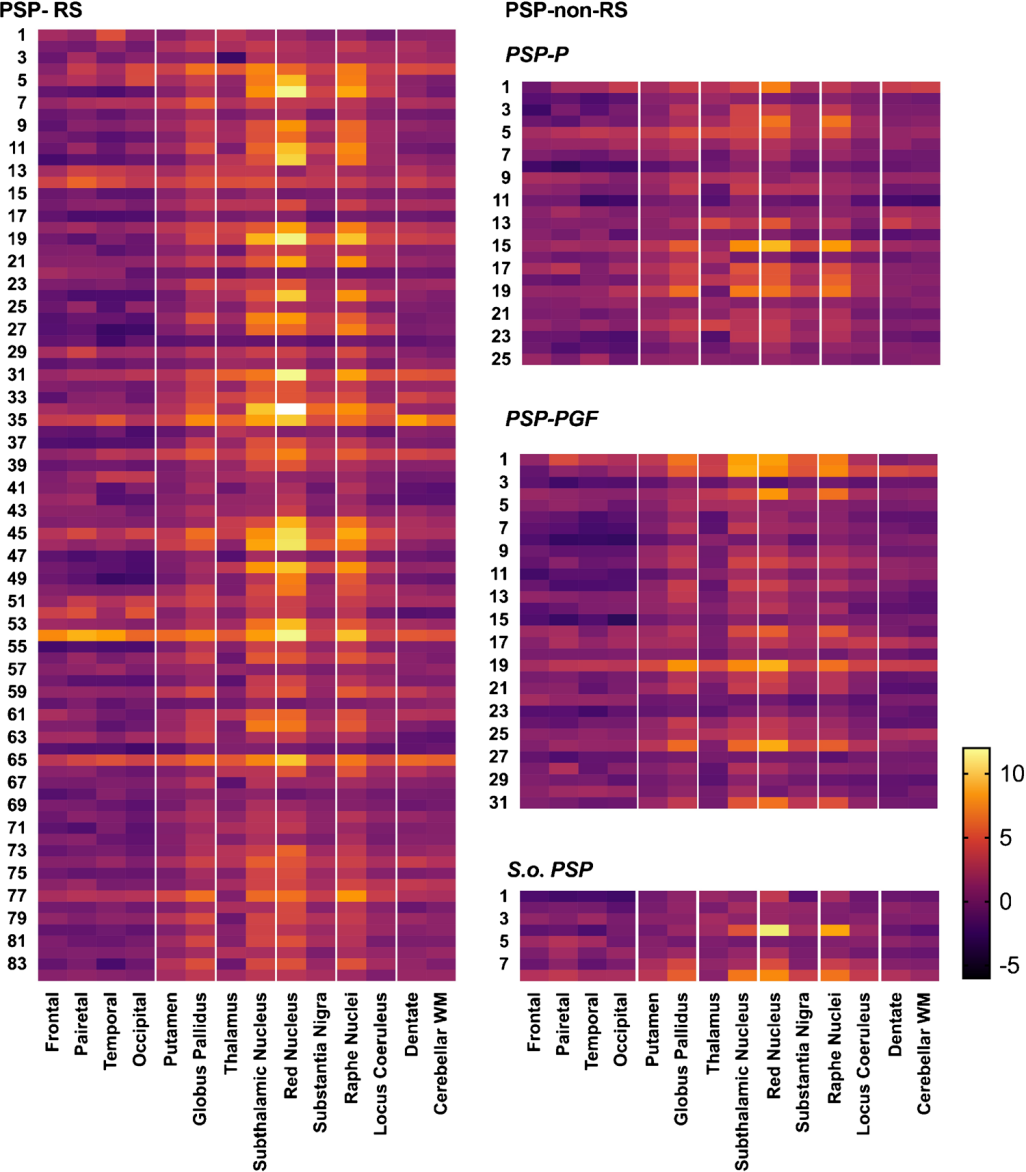
Heat maps for the individual distribution of ^18^F-Florzolotau SUVR Z scores in different progressive supranuclear palsy phenotypes. Abbreviations: PSP, progressive supranuclear palsy; PSP-RS, progressive supranuclear palsy-Richardson’s syndrome; PSP-non-RS, other PSP subtypes expect progressive supranuclear palsy-Richardson’s syndrome; PSP-PGF, progressive supranuclear palsy with predominant gait freezing; S.o. PSP, suggestive of progressive supranuclear palsy; WM, white matter. The color bar indicates SUVR Z scores

Figure 3 presents the heat maps for the individual distribution of ^18^F-Florzolotau SUVR Z scores in different PSP phenotypes, with the highest values being observed for the PSP-RS group. The distribution of ^18^F-Florzolotau retention was found to follow the patterns of tau pathology described in previous post mortem studies [2, 3] (Supplementary Fig. 2).

### Regional vulnerability

**Fig. 4:**
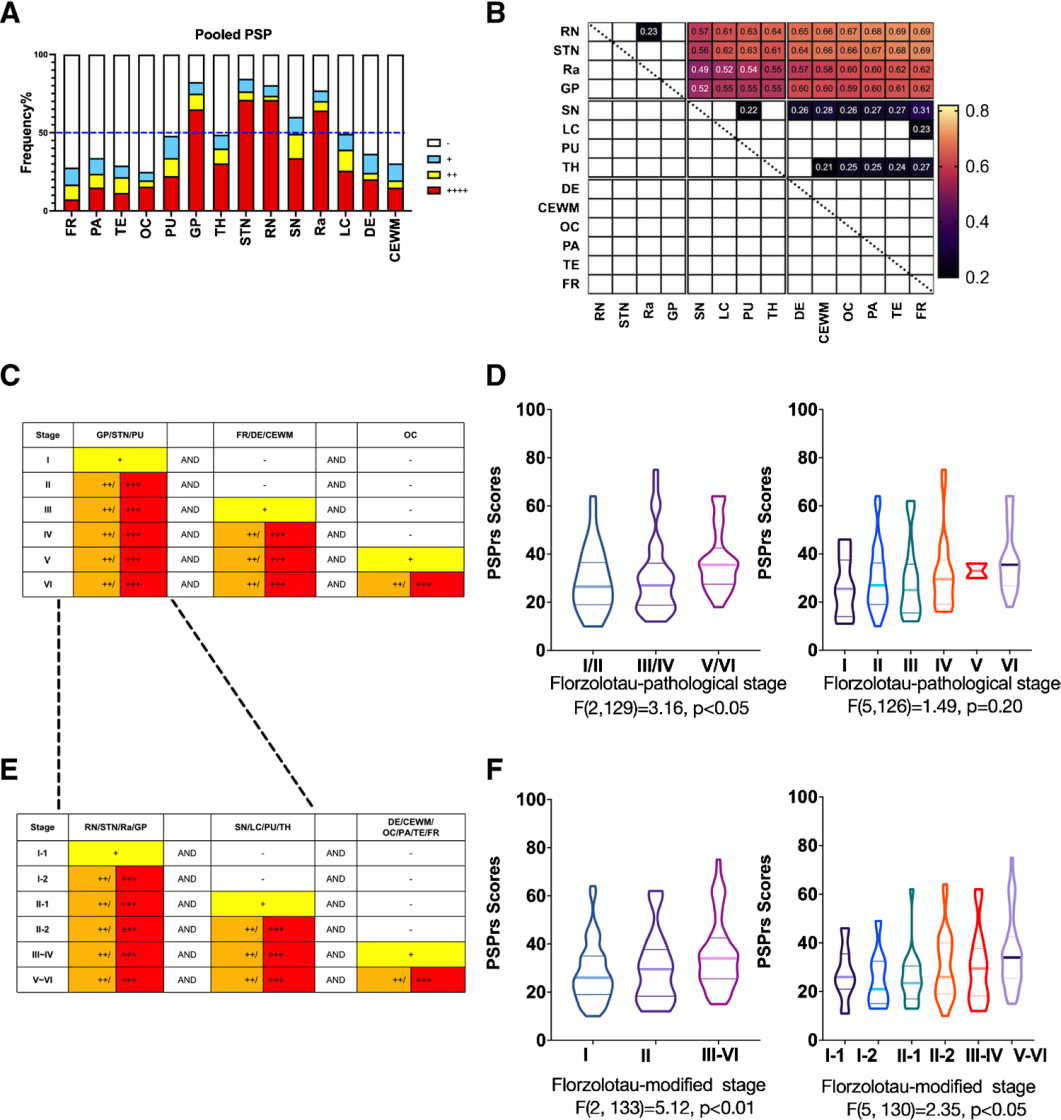
Regional vulnerability and ^18^F-Florzolotau PET staging system for patients with progressive supranuclear palsy. Frequencies of positive regions (panel **A**) and associated conditional probabilities (panel **B**) in the entire study cohort. Criteria for determining the ^18^F-Florzolotau pathology staging system (panel **C**) and the ^18^F-Florzolotau modified staging system (panel **E**). Disease severity was characterized with the PSP rating scale (PSPrs) and analyzed according to disease stages (panel **D** and **F**). Abbreviations: PSP, progressive supranuclear palsy; FR, frontal cortex; PA, parietal cortex; TE, temporal cortex; OC, occipital cortex; PU, putamen; GP, globus pallidus; TH, thalamus; STN, substantia nucleus; RN, red nucleus; SN, substantia nigra; Ra, raphe nuclei; LC, locus coeruleus; DE, dentate; CEWM, cerebellar white matter; -, negative; + , mildly positive; ++ , moderately positive; + + + , strongly positive

When the entire cohort of 148 patients with PSP was analyzed, positive ^18^F-Florzolotau PET findings were more commonly observed in the subthalamic nucleus, red nucleus, globus pallidus, and raphe nuclei (Fig. 4A*)*. The distribution patterns did not show major differences according to PSP phenotypes, although the frequency of positive regions was higher in patients with PSP-RS than in those diagnosed with PSP-non-RS (Supplementary Fig. 3A–D). On analyzing the conditional probability distributions of tau accumulation in the entire study cohort, tracer retention was found to start in Cluster I regions (red nucleus, subthalamic nucleus, raphe nuclei, and globus pallidus) and spread out to Cluster II regions (thalamus, locus coeruleus, substantia nigra and putamen); finally, Cluster III regions (dentate nucleus, cerebral white matter, and cortices) were affected. Similar findings were observed in separate analyses of PSP-RS and PSP-non-RS groups (Supplementary Fig. 3E–F). The heat maps for the spatial patterns of tau accumulation based on the results of conditional probability analysis are shown in Fig. 4B.

### ^18^F-Florzolotau PET staging of PSP

When the pathological staging system proposed in a recent post mortem study [2] was applied in light of ^18^F-Florzolotau PET findings (^18^F-Florzolotau pathological staging system, Fig. 4C), the stage distribution of the 148 patients with PSP was as follows: stage I (*n* = 12, 8.1%), stage II (*n* = 51, 34.5%), stage III (*n* = 20, 13.5%), stage IV (*n* = 22, 14.9%), stage V (*n* = 3, 2.0%), and stage VI (*n* = 28, 18.9%). The remaining 12 patients (8.1%) were not classifiable (Supplementary Fig. 4). Based on the conditional probability distributions of tau accumulation in the current study and the previous pathology staging system [2], we devised a ^18^F-Florzolotau modified staging system (Fig. 4E), in which stage I-1 − I-2 was defined as evidence of ^18^F-Florzolotau binding in the red nucleus, subthalamic nucleus, raphe nuclei, and globus pallidus (Cluster I); stage II-1 − II-2 as ^18^F-Florzolotau accumulation in the substantia nigra, locus coeruleus, putamen, and thalamus (Cluster II); and stage III − VI as ^18^F-Florzolotau binding in the dentate nucleus, cerebral white matter, and cortices (Cluster III). On applying the ^18^F-Florzolotau modified staging system, the following distribution was observed in the study cohort: stage I-1 (*n* = 13, 8.8%), stage I-2 (*n* = 10, 6.8%), stage II-1 (*n* = 18, 12.2%), stage II-2 (*n* = 28, 18.9%), stage III − IV (*n* = 16, 10.8%), and stage V − VI (*n* = 56, 37.8%). The remaining 7 patients (4.7%) were not classifiable (Supplementary Fig. 5). For ^18^F-Florzolotau modified staging system, the clinical severity of PSP, as reflected by PSPrs scores, increased in parallel with disease stages in both three-level (I, II, III-VI; *p* = 0.007) and six-level (I-1, I-2, II-1, II-2, III-IV, V-VI; *p* = 0.044; Fig. 4F) staging, while for ^18^F-Florzolotau pathological staging system such association between clinical severity and staging was only significant in three-level classification (I-II, III-IV, V-VI; *p* = 0.046) (Fig. 4D).

## Discussion

On analyzing a large cohort of patients with PSP across different phenotypes, we found that the distribution patterns of ^18^F-Florzolotau accumulation observed in the living brains of patients with PSP are characterized by a remarkable similarity to the recently reconstructed maps of tau accumulation reported in post mortem research [2]. Moreover, we devised a ^18^F-Florzolotau modified staging system focusing on earlier disease stages which is expected to provide a reliable reflection of in vivo disease progression. Collectively, these data indicate that ^18^F-Florzolotau PET imaging holds promise for tracking the accumulation of tau pathology in the living brain of patients with PSP.

Interestingly, while the patterns of ^18^F-Florzolotau retention in PSP-RS and PSP-non-RS were similar, the binding intensity was markedly lower in the latter group. Tau PET imaging markers have been related to the trajectory of clinical deterioration during disease progression; therefore, the observed differences between the two groups may be a reflection of a varying disease severity. In our study, ^18^F-Florzolotau binding was found to mainly occur within areas belonging to the pallido-nigro-luysian axis — a key area implicated in the pathogenesis of PSP [2]. On post mortem examinations, early pathological accumulation of tau in the pallido-nigro-luysian axis was found to be shared between different clinical PSP phenotypes (i.e., PSP-RS and PSP-non-RS) [2]. Likewise, we demonstrated that ^18^F-Florzolotau deposition progressed with a similar sequence of involvement in PSP-RS and PSP-non-RS, although the former phenotype had a higher burden compared to the latter. These results not only confirmed the validity of previous pilot observations using second-generation PET tau tracers [11, 15], but also supported the existence of specific clinical trajectories in PSP. Specifically, the majority of patients with PSP-non-RS are eventually expected to develop features of PSP-RS [1]. This possibility, which is in line with preliminary pathological observations on tau propagation [4], should be further substantiated in imaging studies with longitudinal follow-up. However, we cannot rule out a role for distinct disease phenotypes — which have been related to a heterogeneous pathological tau distribution. Given that different uncommon clinical phenotypes (e.g., PSP-P and PSP-PGF) were grouped together within the PSP-non-RS group, a certain degree of heterogeneity in terms of tau distribution was expected. It is also worth noting that post mortem studies found that early pathological accumulation of tau in the pallido-nigro-luysian axis was detectable in different clinical PSP phenotypes. Further research is warranted to investigate tau deposition and propagation in larger sample sizes, using both different disease phenotypes and a longitudinal design. In addition, future implementation of deep learning-assisted analyses of tracer uptake is likely to promote standardization of the assessment of tau deposition in different clinical phenotypes, which will allow specific pathophysiological questions related to PSP heterogeneity to be addressed on a larger scale.

We found that the use of ^18^F-Florzolotau — a second-generation tau tracer [8] — allowed tracking regional tau vulnerability as Kovacs et al. [2] did in their post mortem investigation through the use of immunostaining. While the first-generation tau PET tracer ^18^F-Flortaucipir failed to track the neuropathological staging of PSP-related changes [6], possibly because of off-target binding [4], the positive correlation between ^18^F-Florzolotau pathological staging system and clinical severity as per PSPrs score suggest that ^18^F-Florzolotau may serve as a promising biomarker of 4R tau pathology in PSP. The lack of significance when analyzing on the six-level systems may be because of the limited sample size in the advanced stages (i.e., *n* = 3 in stage V). Due to the nature of post mortem research, most of the enrolled patients with PSP were at the advanced stages with relatively diffused tau accumulation. Therefore, the proposed pathological staging system by Kovacs and colleagues [2] paid more attention to cortical regions and cerebellum (stage III–VI) than the earliest involved subcortical areas (stage I–II). Further study with a lager sample size, especially including more patients at later stages was necessary to validate the feasibility of ^18^F-Florzolotau pathological staging system as an in vivo biomarker. Notably, some patients with relatively high ^18^F-Florzolotau SUVR in the occipital lobe were labeled as stage I/II due to the absence of abnormal uptake in stage III–IV brain regions. While this may be partly explained by the methodological issue that the quantification of ^18^F-Florzolotau signal in this area might be overestimated due to the spillover from the surrounding cerebellum, longitudinal imaging to track the tau propagation in vivo as well as in vitro pathological evidence was warranted.

Taking advantage of the in vivo biomarker of tau, the current study was able to learn more about the characteristics of those at early stages. Thus, in an effort to make ^18^F-Florzolotau PET staging of living brains less weighted by the relative importance of brain regions affected in late phases of the disease course, we devised a modified staging system based on conditional probability distributions of tau accumulation. The ^18^F-Florzolotau PET modified staging system with significant association to PSPrs scores harbored high potential for tracking tau deposition in the early stages of the disease course. These findings highlight the anatomical substrates for the trajectory of disease severity and may prompt longitudinal investigations with repeated scans within the same patients. It is worth noting that when assessing the correlation between the revised staging system and clinical severity, the later stages (III–VI) were not subdivided separately, given that this model was specifically aimed at better defining early disease stages as well as the limited enrollment of patients in advanced stages. Besides, there were a small number of patients with PSP who did not meet the proposed staging systems and were considered unclassifiable (8.1% and 4.7% in the ^18^F-Florzolotau pathological and modified staging systems, respectively), which was also reported in previous post mortem studies [2]. Successful validation of ^18^F-Florzolotau PET staging models for PSP will be paramount to assess their diagnostic and predictive utility and to evaluate their usefulness for determining treatment efficacy in clinical trials.

### Strengths and limitations

The assessment of in vivo tau deposition using a second-generation tau tracer sensitive to 4R tau deposits, the systematic analysis of regional abnormalities via ROI-level comparisons, and the large and well-characterized clinical cohort, enabling an analysis of PSP phenotypes, all constitute study strengths. However, several limitations must be noted. First, this is a single-center study and the results clearly require replication. Second, we could not investigate the association between ^18^F-Florzolotau imaging results and post mortem findings; the integration of autopsy assessments with PET imaging represents a continued need that will require harmonized multicenter datasets. Third, the staging system devised in our study was based on cross-sectional results of in vivo ^18^F-Florzolotau PET imaging, and not all cases were classifiable with PET staging. Patients with PSP should be followed longitudinally in relation to the propagation patterns to evaluate if this classification can provide prognostic information. Fourth, the control group was relatively small and not-well matched to the patient group in demographics. Further validation with more controls should be conducted. Finally, it would have been interesting to analyze the correlation between tau deposition and other pathological changes (e.g., astrogliosis, dopaminergic dysfunction, and neurodegeneration); however, as this was not planned while the study was being designed, we are unable to provide these data. The question as to whether multiple imaging modalities, combined by an integrative analytical approach, could have a higher predictive accuracy for PSP should be addressed in future studies.

## Conclusions

^18^F-Florzolotau PET imaging can effectively reflect the characteristic accumulation of tau pathology in PSP. The ^18^F-Florzolotau PET modified staging system allowed early tracking of tau deposition and disease severity assessment. Patients with PSP should be followed longitudinally in relation to the distribution patterns to evaluate whether this classification may inform prognosis.

## Supplementary Information

Below is the link to the electronic supplementary material.

Supplementary file1 (DOC 9925 KB)

## Data Availability

The datasets generated during and/or analyzed during the current study are available from the corresponding authors (wangjian_hs@fudan.edu.cn OR zuochuantao@fudan.edu.cn) on reasonable request.
