# Supplementary material for: 18F-Florzolotau PET imaging captures the distribution patterns and regional vulnerability of tau pathology in progressive supranuclear palsy
Source: Eur J Nucl Med Mol Imaging. 2023 Jan 11;50(5):1395–405. doi: 10.1007/s00259-022-06104-0 (PMC10027831; doi:10.1007/s00259-022-06104-0)
Supplement: Supplementary file 1 — Supplementary file1 (DOC 9925 KB) [file 259_2022_6104_MOESM1_ESM.doc]

**Supplementary Materials**

**Supplementary Methods.** Methodology used for conditional probability analysis

**Supplementary Figure 1.** ^18^F-Florzolotau PET images in different PSP subtypes

**Supplementary Figure 2.** The distribution of ^18^F-Florzolotau retention in patients with progressive supranuclear palsy followed the patterns of tau pathology previously reported in *post mortem* studies

**Supplementary Figure 3.** Positivity ratios for various brain regions in patients with different progressive nuclear palsy phenotypes and associated conditional probabilities

**Supplementary Figure 4.** Individual regional SUVR Z distributions and ^18^F-Florzolotau pathological staging

**Supplementary Figure 5.** Individual regional SUVR Z distributions and ^18^F-Florzolotau modified staging

**Supplementary Table 1.** General characteristics of the study patients according to different disease phenotypes

**Supplementary Table 2.** Comparison of ^18^F-Florzolotau binding between different PSP subtypes: voxel-level analysis

**Supplementary Table 3.** Comparisons of regional standardized uptake value ratio values according to different disease phenotypes

**Supplementary Methods**

**Methodology used for conditional probability analysis**

The analysis was conducted in a stepwise fashion. First, SUVR values in each examined region were calculated by taking the cerebellar grey matter as reference. Second, SUVR Z scores were calculated using data from the healthy controls. Third, a score of 1 was assigned for strongly positive regions (SUVR Z score ≥ 2); conversely, a score of 0 was given for negative, mildly positive, or moderately positive regions (SUVR Z score < 2). Conditional probability analysis was subsequently applied to determine the patterns of tau accumulation in different anatomical regions, taking into account of a feasible spatial evolution for different regions.

On performing conditional probability analysis, two regions were compared for discordance using all possible combinations. It was therefore possible that one region was affected (score = 1) and the other was not (score = 0). The null hypothesis was that the probability of region A being positive (score = 1) while region B being negative (score = 0) and the region A being negative (score = 0) and region B being positive (score = 1) would be equally likely; thus, the A and B regions were considered to be simultaneously affected. This approach allowed assigning a conditional probability that one region would be affected by tau accumulation before another one. The McNemar’s test was applied to measure the strength of the evidence against the null hypothesis. We generated a matrix for tau depositions involving various anatomical regions; in this analysis, each cell in the matrix corresponded to a conditional probability that one region was involved before another. Conditional probability was calculated using the crosstab function available in SPSS (version 22.0; IBM, Armonk, NY, USA) [2].

| Region A | Region B |  |
| --- | --- | --- |
|  | Affected, Score 1 | Not affected, score 0 |
| Affected, score 1 | W | X |
| % of cases within region A | % | % |
| % of cases within region B | % | Conditional probability A preceding B |
| Not affected, score 0 | Y | Z |
| % of cases within region A | Conditional probability B preceding A | % |
| % of cases within region B | % | % |

When the conditional probability for one region was significantly higher than that of another region, the former was deemed more likely to be affected than the latter. When the conditional probability for one region was ≥ 0.20 and the p value was < 0.01, the region was considered as more likely affected prior to the other.

**Supplementary Figure 1**

**
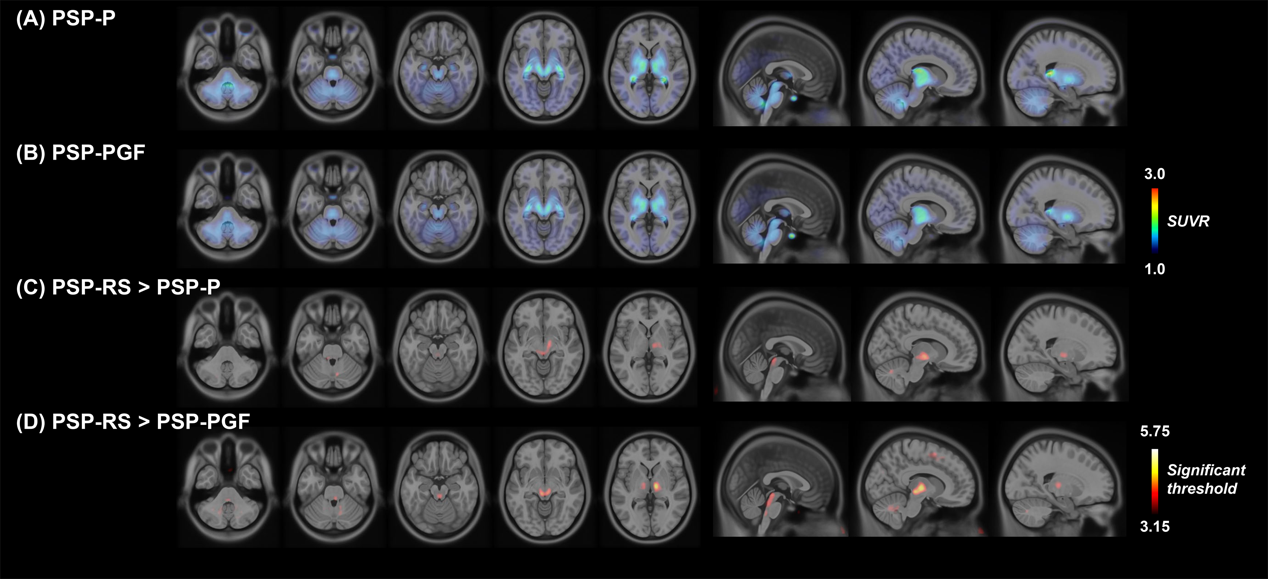
**

**Supplementary Figure 1. ^18^F-Florzolotau PET images in different PSP subtypes**

^18^F-Florzolotau PET images in progressive supranuclear palsy with predominant parkinsonism (PSP-P) (panel A) and progressive supranuclear palsy with predominant gait freezing (PSP-PGF) (panel B); comparison of ^18^F-Florzolotau binding between progressive supranuclear palsy-Richardson’s syndrome (PSP-RS) and PSP-P (panel C); comparison of ^18^F-Florzolotau binding between PSP-RS and PSP-PGF (panel D). Abbreviation: SUVR, standardized uptake value ratio.

**Supplementary Figure 2**

**
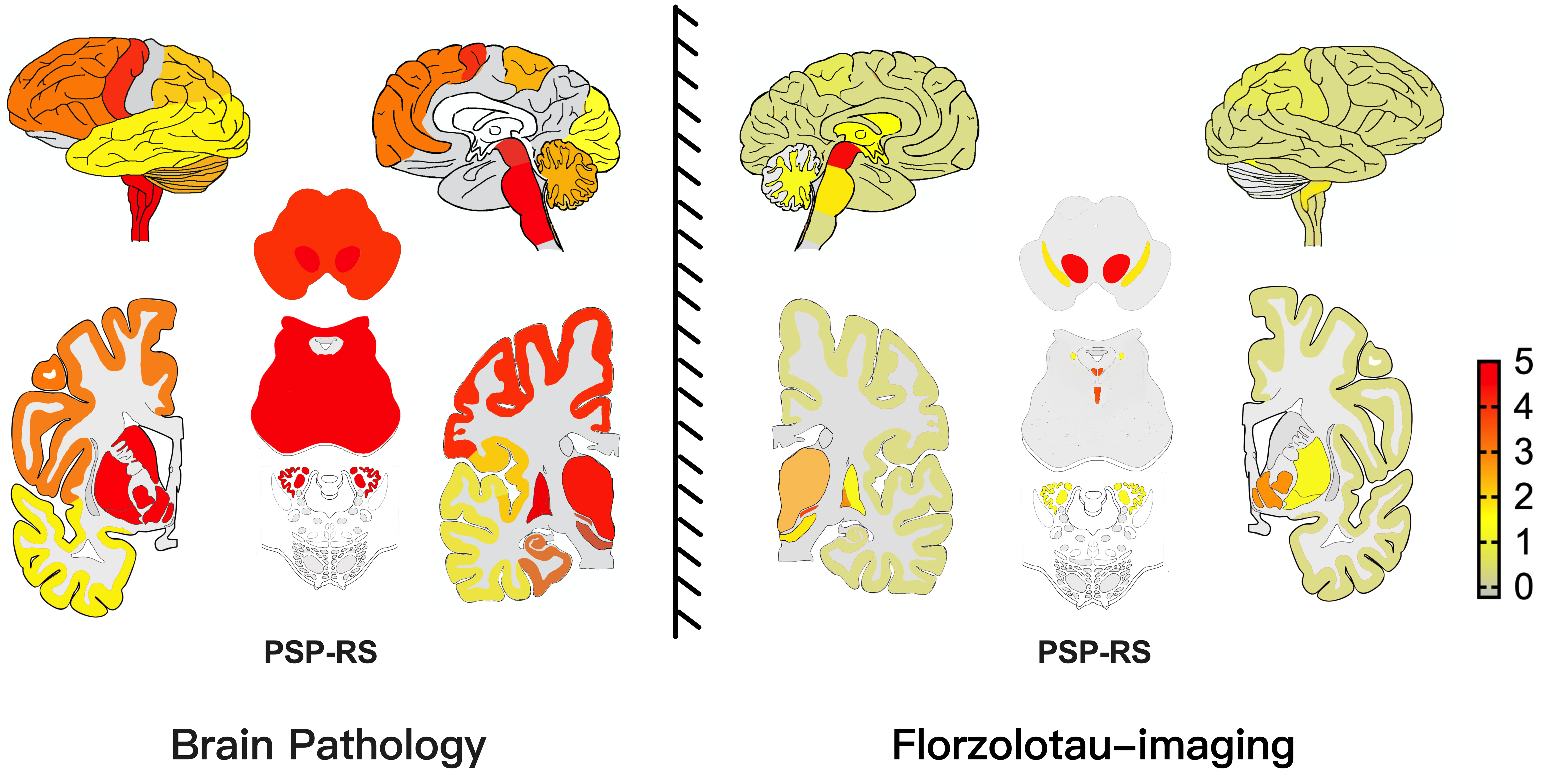
**

**Supplementary Figure 2. The distribution of ^18^F-Florzolotau retention in patients with progressive supranuclear palsy followed the patterns of tau pathology previously reported in *post mortem* studies**

Abbreviation: PSP-RS, progressive supranuclear palsy-Richardson’s syndrome. The color bar indicates the SUVR Z scores.

**Supplementary Figure 3**

**
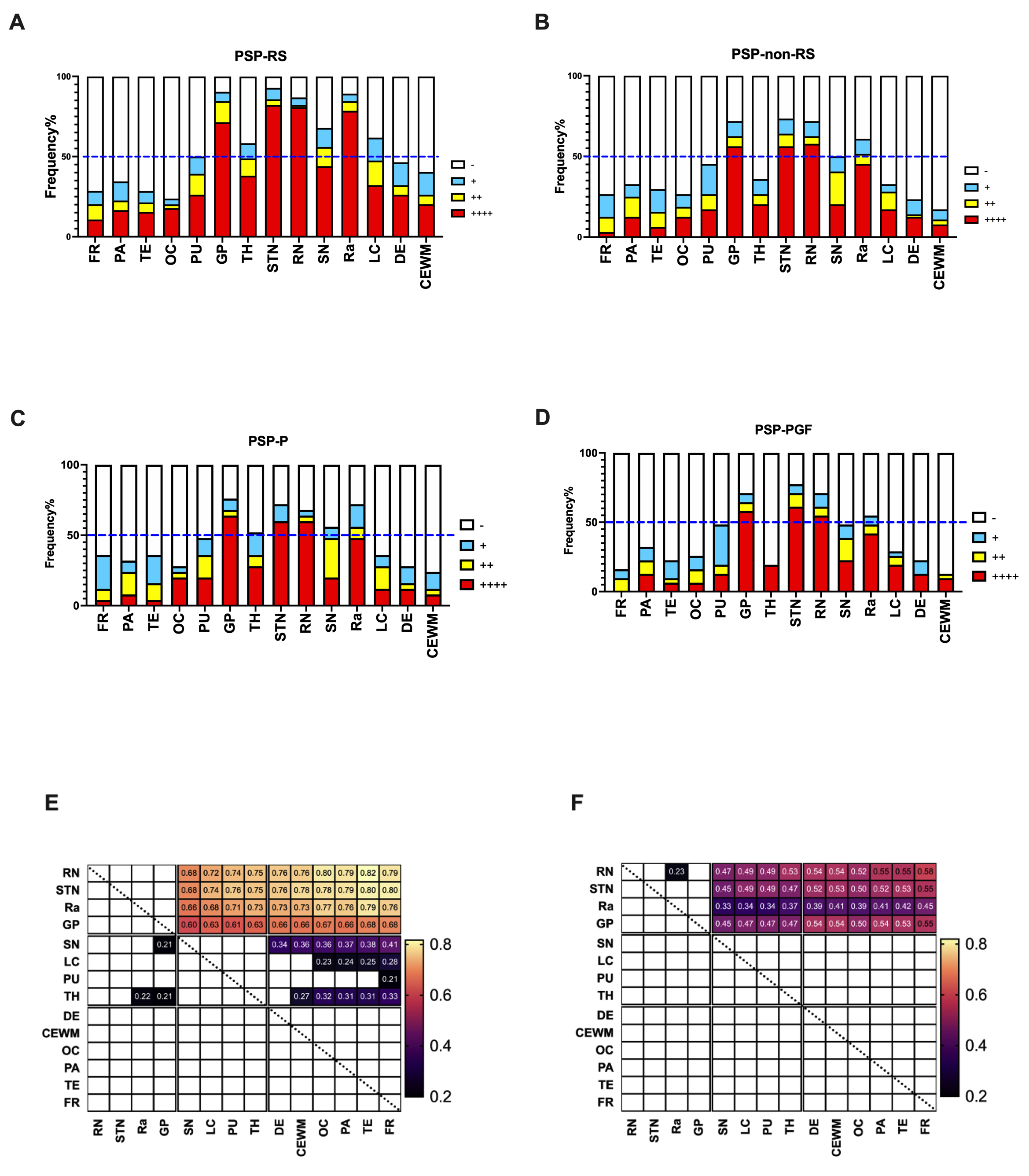
**

**Supplementary Figure 3. Positivity ratios for various brain regions in patients with different progressive nuclear palsy phenotypes and associated conditional probabilities**

Positivity ratios for various brain regions in patients with different progressive nuclear palsy phenotypes (panels A-D). The associated conditional probabilities in PSP-RS and PSP-non-RS are shown in panels E and F. Abbreviations: PSP, progressive supranuclear palsy; PSP-RS, progressive supranuclear palsy-Richardson’s syndrome; PSP-non-RS, other PSP subtypes with the exception of progressive supranuclear palsy-Richardson’s syndrome; PSP-P, progressive supranuclear palsy with predominant parkinsonism; PSP-PGF, progressive supranuclear palsy with predominant gait freezing. FR, frontal cortex; PA, parietal cortex; TE, temporal cortex; OC, occipital cortex; PU, putamen; GP, globus pallidus; TH, thalamus; STN, substantia nucleus; RN, red nucleus; SN, substantia nigra; Ra, raphe nuclei; LC, locus coeruleus; DE, dentate; CEWM, cerebellar white matter; -, negative; +, mildly positive; ++, moderately positive; +++, strongly positive.

**Supplementary Figure 4**

**
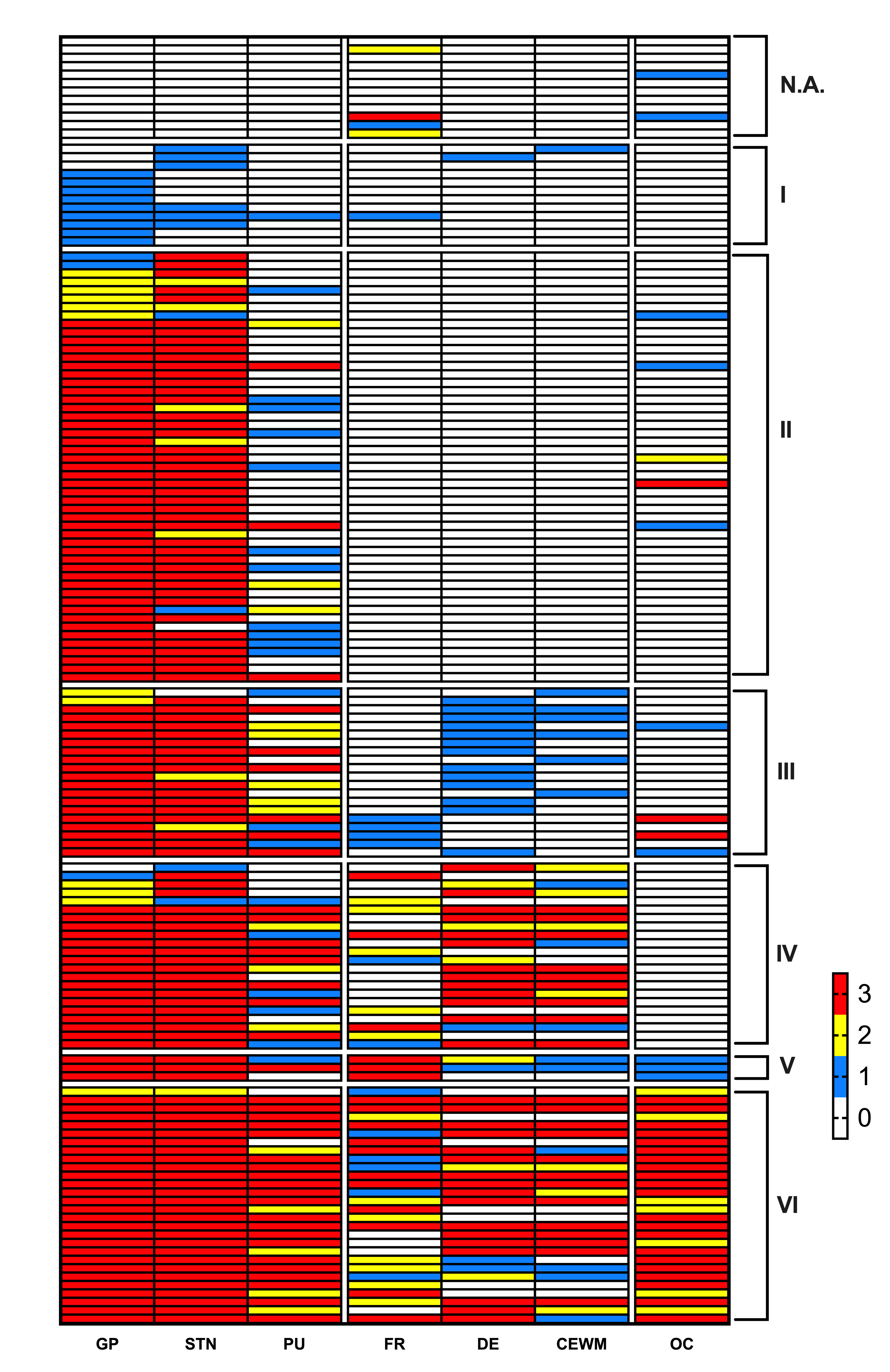
**

**Supplementary Figure 4. Individual regional SUVR Z distributions and ^18^F-Florzolotau pathological staging**

0 = “-”, 1 = “+”, 2 = “++”, and 3 = “+++” indicate negative, mild, moderate, and strong involvement, respectively. Abbreviations: GP, globus pallidus; STN, substantia nucleus; PU, putamen; FR, frontal cortex; DE, dentate; CEWM, cerebellar white matter; OC, occipital cortex; N.A., not applicable.


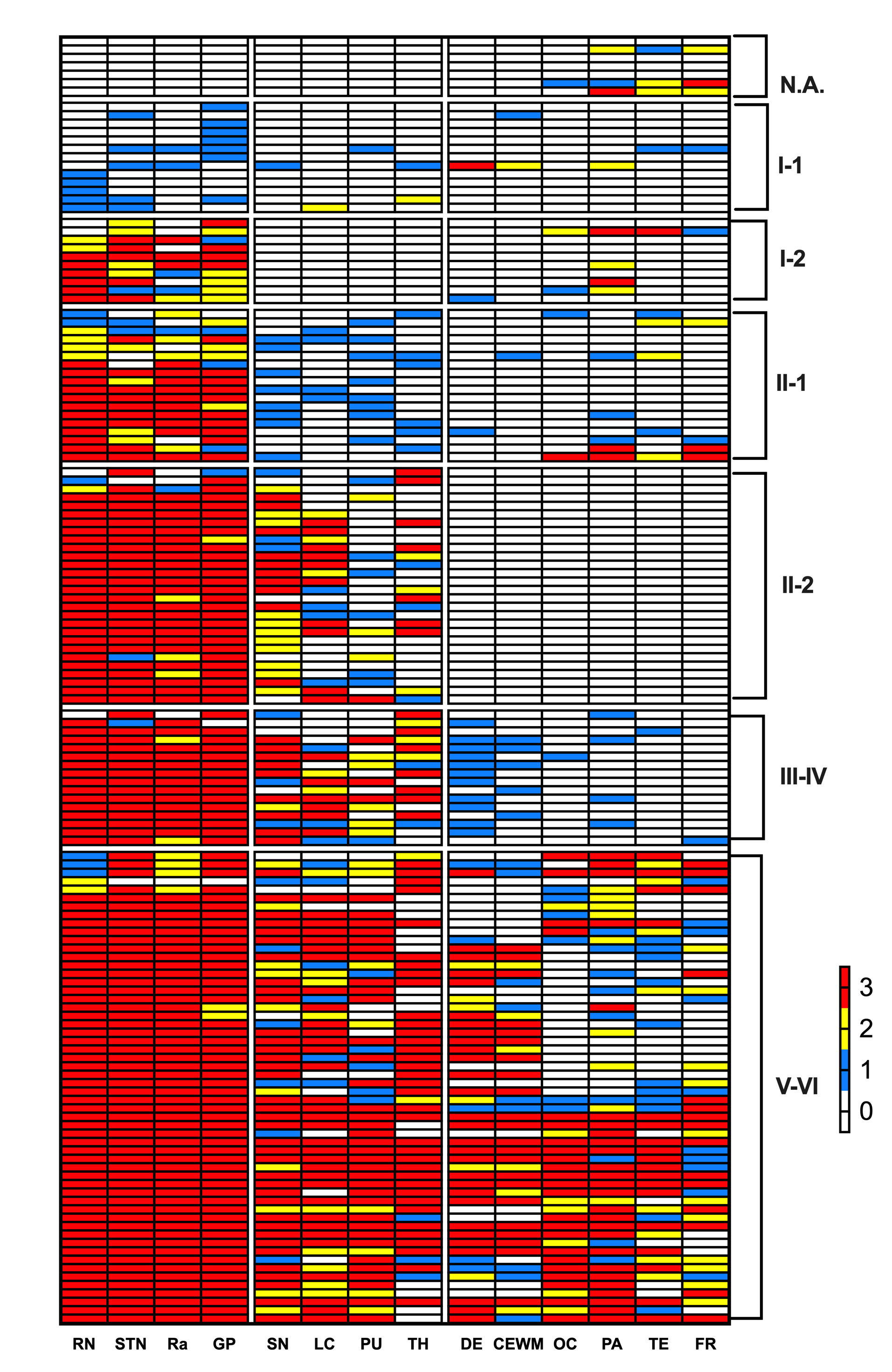
**Supplementary Figure 5**

**Supplementary Figure 5. Individual regional SUVR Z distributions and ^18^F-Florzolotau modified staging**

0 = “-”, 1 = “+”, 2 = “++”, and 3 = “+++” indicate negative, mild, moderate, and strong involvement, respectively. Abbreviations: RN, red nucleus; STN, substantia nucleus; Ra, raphe nuclei; GP, globus pallidus; SN, substantia nigra; LC, locus coeruleus; PU, putamen; TH, thalamus; DE, Dentate; CEWM, cerebellar white matter; OC, occipital cortex; PA, parietal cortex; TE, temporal cortex; FR, frontal cortex; N.A., not applicable.

**Supplementary Table 1. General characteristics of the study patients according to different disease phenotypes**

|  | **PSP-RS** | **PSP-P** | **PSP-PGF** | ***S.o. PSP*** | **p** |
| --- | --- | --- | --- | --- | --- |
| Number of subjects | 84 | 25 | 31 | 8* | / |
| Sex (men/women) | 43/41 | 17/8 | 20/11 | 3/5 | 0.23^b^ |
| Age (years) | 66.3 ± 5.8 | 65.4 ± 7.0 | 65.6 ± 8.0 | 61.0 ± 9.4 | 0.39^c^ |
| Age at onset (years) | 63.0 ± 6.2 | 60.1 ± 7.7 | 62.2. ± 7.8 | 57.1 ± 9.3 | 0.08^a^ |
| Disease duration (months) | 38.0  (24.0, 52.0)^##^ | 70.0  (18.5, 99.5) | 35.0  (17.0, 61.0)^#^ | 29.0  (17.5, 37.0)^#^ | 0.06^c^ |
| PSPrs score | 32.0  (22.0, 43.0) | 26.0  (24.0, 35.0) | 22.0  (14.5, 28.0)*** | 13.0  (12.0, 22.0)* | <0.01^c^ |
| MMSE score | 24 (19, 27) | 25 (23, 28) | 27 (25, 28)* | 27 (21, 28) | 0.01^c^ |
| Education (years) | 9.0  (6.0, 12.0) | 12.0  (9.0, 13.3) | 10.0  (7.0, 12.0) | 10.0  (7.5, 14.3) | 0.20^c^ |
| LEED (mg) | 400.0  (150.0, 600.0) | 400.0  (175.0, 675.0) | 400.0  (300.0, 650.0) | 375  (75.0, 600.0) | 0.95^c^ |

Data are expressed as means ± standard deviations or medians (interquartile ranges). Abbreviations: PSP, progressive supranuclear palsy; PSP-RS, progressive supranuclear palsy-Richardson’s syndrome; PSP-P, progressive supranuclear palsy with predominant parkinsonism; PSP-PGF, progressive supranuclear palsy with predominant gait freezing; S.o. PSP, suggestive-of-progressive supranuclear palsy; PSPrs, progressive supranuclear palsy rating scale; MMSE, Mini-Mental State Examination; LEDD, L-dopa equivalent daily dose.

^a^One-way ANOVA; ^b^Chi-square test; ^c^Kruskal-Wallis test. The conservative Bonferroni’s correction was applied to correct for multiple comparisons.

#p < 0.05; ##p < 0.01 *versus* PSP-P; *p < 0.05; ***p < 0.001 *versus* PSP-RS.

**Supplementary Table 2.** Comparison of ^18^F-Florzolotau binding between different PSP subtypes: voxel-level analysis

|  | Cluster size  (mm^3^) | FDR-corrected  P value at  cluster level | Peak T value | Peak coordinate  (X, Y, Z) | Brain regions |
| --- | --- | --- | --- | --- | --- |
| **PSP > Healthy controls** | 35080 | <0.001 | 7.91 | -22, -4, 0 | Left lateral globus pallidus |
|  |  |  | 7.34 | 2, -30, -10 | Left midbrain  (red nucleus) |
|  |  |  | 7.34 | 24, -6, 0 | Right lateral globus pallidus |
| **PSP-RS > PSP-non-RS** | 29240 | <0.001 | 5.80 | -10, -12, 0 | Left thalamus |
|  |  |  | 5.42 | -8, -24, -6 | Left midbrain  (red nucleus) |
|  |  |  | 5.28 | 2, -30, -12 | Left midbrain  (red nucleus) |
|  | 8264 | <0.001 | 4.37 | -24, -62, -38 | Left cerebellum  (cerebellar tonsil) |
|  |  |  | 4.34 | -8, -66, -30 | Left cerebellum  (nodule) |
|  |  |  | 4.02 | -20, -70, -36 | Left cerebellar tonsil |
|  | 3264 | 0.021 | 4.09 | -6, 16, 50 | Left medial frontal gyrus  (Brodmann area 6) |
|  |  |  | 4.04 | 4, -8, 58 | Right medial frontal gyrus  (Brodmann area 6) |
|  |  |  | 3.92 | -10, 10, 56 | Left medial frontal gyrus  (Brodmann area 6) |
| **PSP-RS > PSP-P** | 7688 | <0.001 | 4.43 | -12, -10, -6 | Left midbrain  (Subthalamic nucleus) |
|  |  |  | 4.01 | 0, -30, -10 | Left midbrain  (Red nucleus) |
|  |  |  | 3.82 | -8, -24, -6 | Left midbrain  (Red nucleus) |
| **PSP-RS > PSP-PGF** | 11264 | <0.001 | 4.95 | -10, -14, 0 | Left thalamus |
|  |  |  | 4.78 | -8, -24, -4 | Left thalamus |
|  |  |  | 4.47 | 6, -28, -10 | Right midbrain  (Red nucleus) |
| **PSP-P vs PSP-PGF** | None |  |  |  |  |

Voxel-level two-sample Student’s *t*-tests were performed using SUVR images after adjusting for age, sex, and disease duration (if applicable). Results were considered significant at a voxel-wise p < 0.001 (uncorrected) and a cluster-wise p < 0.05 [false discovery rate correction (FDR) for multiple comparisons]. The peak coordinates are referred to the Montreal Neurological Institute (MNI) brain coordinate system. Abbreviations: PSP, progressive supranuclear palsy; PSP-RS, progressive supranuclear palsy-Richardson’s syndrome; PSP-non-RS, other PSP subtypes with the exception of progressive supranuclear palsy-Richardson’s syndrome; PSP-P, progressive supranuclear palsy with predominant parkinsonism; PSP-PGF, progressive supranuclear palsy with predominant gait freezing; FDR, false discovery rate; MNI, Montreal Neurological Institute.

**Supplementary Table 3. Comparisons of regional standardized uptake value ratio values according to different disease phenotypes**

|  | **PSP-RS**  **(n = 84)** | **PSP-P**  **(n = 25)** | **PSP-PGF**  **(n = 31)** | **p ^a^** | **p ^b^** | **p ^c^** |
| --- | --- | --- | --- | --- | --- | --- |
| **Frontal cortex** | 0.91 ± 0.10 | 0.90 ± 0.08 | 0.88 ± 0.08 | 0.97 | 0.29 | 0.29 |
| **Parietal cortex** | 0.97 ± 0.10 | 0.94 ± 0.08 | 0.93 ± 0.10 | 0.29 | 0.24 | 0.87 |
| **Temporal cortex** | 1.03 ± 0.12 | 1.02 ± 0.09 | 0.99 ± 0.09 | 0.87 | 0.28 | 0.22 |
| **Occipital cortex** | 1.10 ± 0.11 | 1.09 ± 0.09 | 1.07 ± 0.09 | 0.70 | 0.32 | 0.56 |
| **Putamen** | 1.29 ± 0.17 | 1.28 ± 0.13 | 1.23 ± 0.15 | 0.54 | 0.13 | 0.45 |
| **Globus pallidus** | 1.62 ± 0.21 | 1.52 ± 0.20 | 1.55 ± 0.24 | 0.05 | 0.13 | 0.56 |
| **Thalamus** | 1.66 ± 0.28 | 1.59 ± 0.28 | 1.47 ± 0.25 | 0.43 | <0.01 | 0.43 |
| **Subthalamic nucleus** | 1.86 ± 0.29 | 1.68 ± 0.26 | 1.70 ± 0.30 | <0.01 | 0.01 | 0.28 |
| **Red nucleus** | 1.77 ± 0.30 | 1.61 ± 0.22 | 1.60 ± 0.26 | 0.01 | <0.01 | 0.46 |
| **Substantia nigra** | 1.54 ± 0.20 | 1.46 ± 0.19 | 1.47 ± 0.22 | 0.03 | 0.14 | 0.35 |
| **Raphe nuclei** | 1.74 ± 0.28 | 1.59 ± 0.25 | 1.55 ± 0.27 | <0.01 | <0.01 | 0.70 |
| **Locus coeruleus** | 1.47 ± 0.19 | 1.36 ± 0.15 | 1.36 ± 0.19 | <0.01 | <0.01 | 0.46 |
| **Dentate nucleus** | 1.47 ± 0.20 | 1.37 ± 0.15 | 1.34 ± 0.16 | 0.04 | <0.01 | 0.97 |
| **Cerebellar white matter** | 1.35 ± 0.13 | 1.30 ± 0.11 | 1.27 ± 0.12 | 0.11 | 0.01 | 0.87 |

*P* values for pairwise comparisons between the four study groups were adjusted for multiple comparisons using the Benjamini-Hochberg procedure. a, PSP-RS *versus* PSP-P; b, PSP-RS *versus* PSP-PGF; c, PSP-P *versus* PSP-PGF. Abbreviations: PSP, progressive supranuclear palsy; PSP-RS, progressive supranuclear palsy-Richardson’s syndrome; PSP-P, progressive supranuclear palsy with predominant parkinsonism; PSP-PGF, progressive supranuclear palsy with predominant gait freezing.

**Reference**

1. Rolls ET, Huang CC, Lin CP, Feng J, Joliot M. Automated anatomical labelling atlas 3. Neuroimage. 2020;206:116-189.
